# Immune Responses to Nanomaterials for Biomedical Applications

**DOI:** 10.3390/nano11051241

**Published:** 2021-05-08

**Authors:** Giuseppe Bardi

**Affiliations:** Nanobiointeractions & Nanodiagnostics, Istituto Italiano di Tecnologia, Via Morego 30, 16163 Genova, Italy; giuseppe.bardi@iit.it; Tel.: +39-010-289-6519

## Editorial

The present Special Issue hosts six research papers and five review articles regarding different aspects of nanotechnologies for therapeutic and diagnostic applications.

Much attention has been given to the interaction between nanomaterials (NMs) and the innate immune system. Opsonization and phagocytosis are the first natural defenses that our bodies use with foreign particles, either natural or synthetic.

An interesting review article of the Issue by Dukhinova and colleagues [1] discusses the impact that metallic nanoparticles (NPs) have on macrophages, which are key phagocytes of innate immunity that also regulate the fate of the adaptive response. The manuscript reports the molecular mechanisms leading to differentiated macrophage phenotypes in the presence of several metallic NPs and the following inflammatory responses. On the same topic, a research paper by Vasilichin et al. shows data on the effects that these types of particles have on the expression of pattern recognition receptors (PRRs) in human monocytic THP-1, specifically the Toll-Like-Receptors (TLRs) 4 and 6 [2].

Together with monocyte/macrophages, neutrophils are the other phagocytes engulfing NPs released in the bloodstream. Actually, they are the faster innate interceptors. Bilyy and colleagues point out size and surface properties of non-degradable NPs that induce neutrophils’ reactions and the formation of neutrophil extracellular traps (NETs) [3]. General approaches to accurately study immune interactions of metal–NPs are described in the review from Gatto and Bardi [4].

Novel therapeutics often exploit the advantages that NMs can provide. The use of biodegradable materials is commonly welcome for this purpose, as the possibility to transport and release drugs by non-persistent nano-carriers avoids prolonged immune reactions. However, even biodegradable NPs could induce immune reactions due to the intrinsic properties of the chosen material and the surface chemistry of the particle. A broad overview on the synthesis of liposomes considering toxicological and immune aspects is provided by Inglut and colleagues in a comprehensive review article [5]. The authors analyze liposome size, lipid composition, PEGylation, surface charge and their immune consequences once released. To underline the importance of such topics, it is worth mentioning that the anti-SarsCov2 vaccine formulation recently launched by Pfizer to combat the current COVID-19 pandemic contains a PEGylated liposome entrapping the mRNA encoding for the SarsCov2 capsid-protein Spike2 [6]. PEGylation, in particular, raises concerns for potential allergic reactions [7,8].

As well as lipid-based NPs, other biodegradable polymer particles are under deep investigation by the biomedical research community. Especially, NPs synthesized using FDA-approved polymers such as poly(lactide acid) (PLA), and poly(lactic-co-glycolic acid) (PLGA). PLGA NPs loaded with indocyanine green, a photothermal agent (Nexturastat A) and an epigenetic drug to combine photothermal therapy with epigenetic therapy aimed at melanoma type of cancer are described here by Ledezma et al. [9]. Furthermore, the results shown by Duncan and collaborators demonstrate promising opportunities to contrast inflammation using interleukin-10 (IL-10) loaded PLA NPs [10]. They proved that NP-mediated prolonged release (i.e., two months) of the anti-inflammatory cytokine IL-10 was able to modulate the expression of pro-inflammatory cytokines by previously activated mouse macrophages with chlamydial stimulants.

The combination of immune attractant proteins (i.e., chemokines) and PLGA NP-features has been used by our group to abrogate CXCL12-mediated THP-1 migration as shown in Pisani et al. [11]. We believe that this intriguing functionalization of PLGA-NP surfaces with entire chemokines could lead to future therapeutic carriers able to selectively target immune cell subsets. Chemokine decoration of the surface allows the NP to bind to their cognate chemokine-receptors (CCRs/CXCRs). The receptor expression on the cell membrane depends on the cell type as well as its physiological/pathological state. Then, the chemokine choice defines the target, whereas the NP could impair the natural functions of the free chemokine. In this work, we stopped cell migration using CXCL12-PLGA NPs. Due to the relevance of this chemokine and its receptor (i.e., CXCR4) in immune cell homeostasis and cancer metastasis, we hope to contribute to future nano-carriers to be applied for research and therapeutic purposes.

The issue also includes a manuscript regarding predictions of hyper-thermic cell death by adopting a “three-state” mathematical model coupled with a tumor growth model [12]. The proposed in silico methodology and some preliminary results could be considered an important starting point to accurately simulate the hyperthermia-based tumor control also involving the immune system.

Last but not least, a methodology paper on the use Transmission Electron Microscopy (TEM) to carefully study the effects of ionizing radiation (IR) combined to gold NPs has been included [13]. The authors provide valuable insights on the NPs’ radio-sensitization and the subsequent biochemical mechanisms through immunogold-labelling of antigenic sites at ultrastructural level.

As previously mentioned by the relevant information of the published papers, this Special Issue highlights the importance of host immune responses to NMs as a fundamental subject to be addressed in order to create safe-by-design NPs for therapeutic and diagnostic applications in biomedicine.
